# Supplementary figures and images for: PHRF1 promotes migration and invasion by modulating ZEB1 expression
Source: PLoS One. 2020 Jul 30;15(7):e0236876. doi: 10.1371/journal.pone.0236876 (PMC7392320; doi:10.1371/journal.pone.0236876)

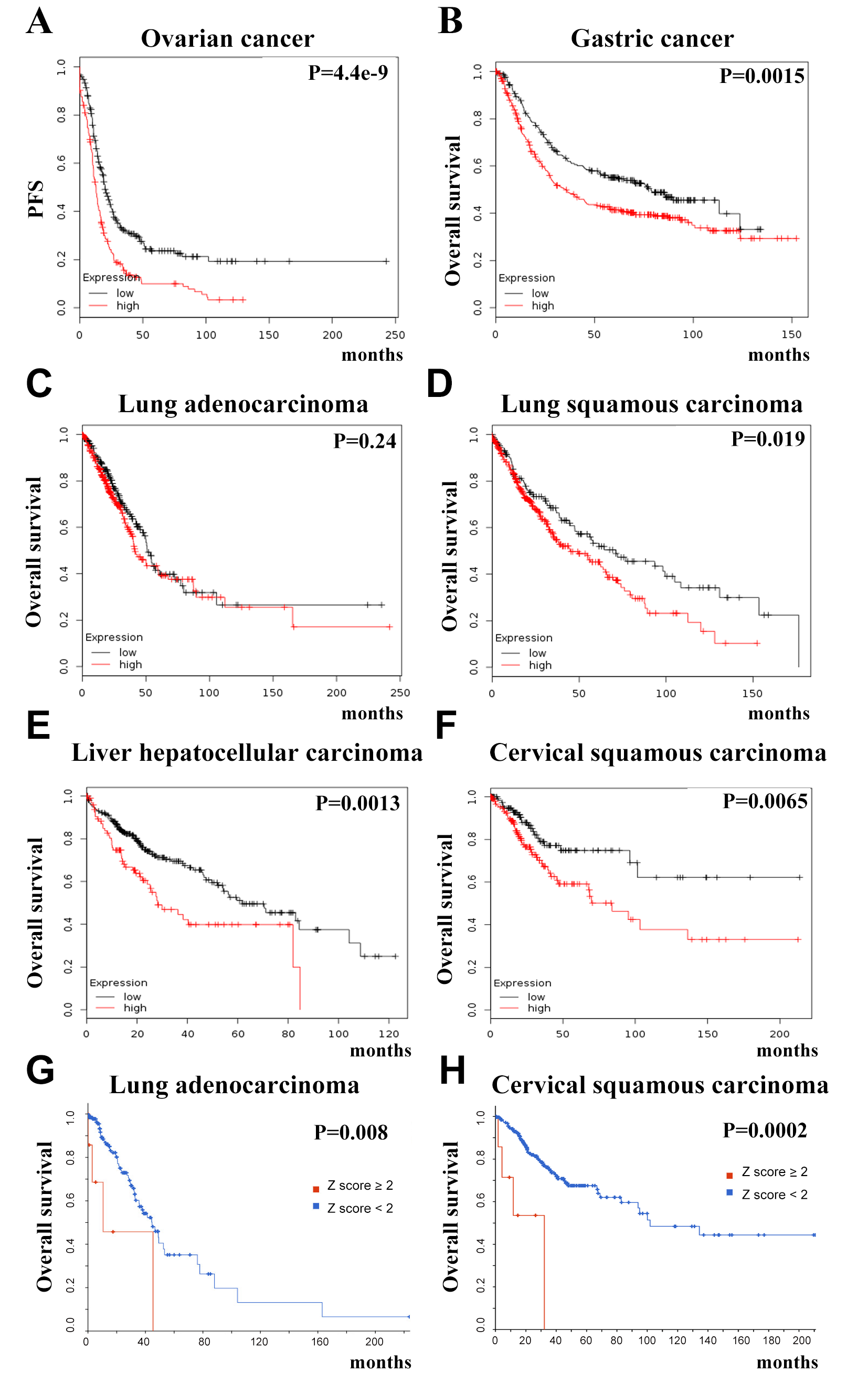

Supplement: S1 Fig — (A) Ovarian cancer. n = 1436. (B) Gastric cancer. n = 882. (C) Lung adenocarcinoma. n = 513. (D) Lung squamous carcinoma. n = 501. (E) Liver hepatocellular carcinoma. n = 371. (F) Cervical squamous carcinoma. n = 304. The cohorts were divided into two groups, high (red) and low (black), according to the median expression value of PHRF1, which were retrieved from the Kaplan–Meier plotter database (http://kmplot.com/analysis/index.php?p=service&cancer). (G) Lung adenocarcinoma. n = 203. (H) Cervical squamous carcinoma. n = 275. Patient data were obtained from cBioPortal TCGA Nature 2014 and TCGA PanCancer Atlas datasets, respectively. Z score ≥ 2 (red). (TIF) [file pone.0236876.s001.tif]

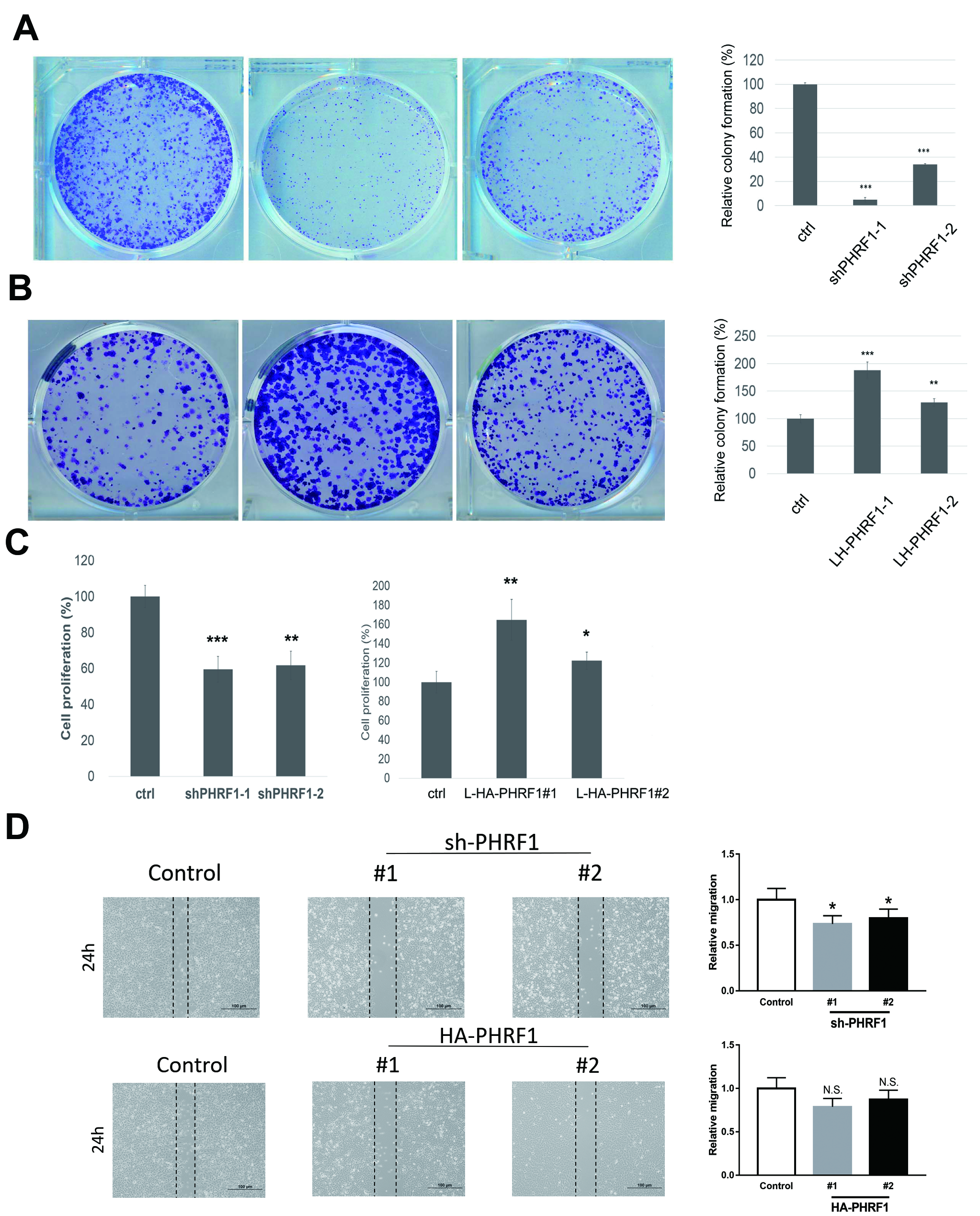

Supplement: S2 Fig — (A) Knockdown of PHRF1 with two specific siRNAs, and (B) overexpression of PHRF1 with two lentiviral transductions, were carried out in lung cancer A549 cells. Anchorage-dependent colony formation was examined for 12 days of culture and then stained with crystal violet. Colonies larger than 0.1 mm in diameter were scored. Quantitative results are shown in the right panels. (C) Cell proliferation was measured using a BrdU colorimetric assay (Roche Diagnostics, Mannheim, Germany). Each bar represents the mean ± SD of three independent experiments. (**P < 0.01 and ***P < 0.001 compared with the controls). (D) PHRF1-depleted and -overexpressing A549 cells were subjected to wound healing assay for 24 h in 0.5% FBS culture medium. Scale bar, 0.1 mm. (TIF) [file pone.0236876.s002.tif]

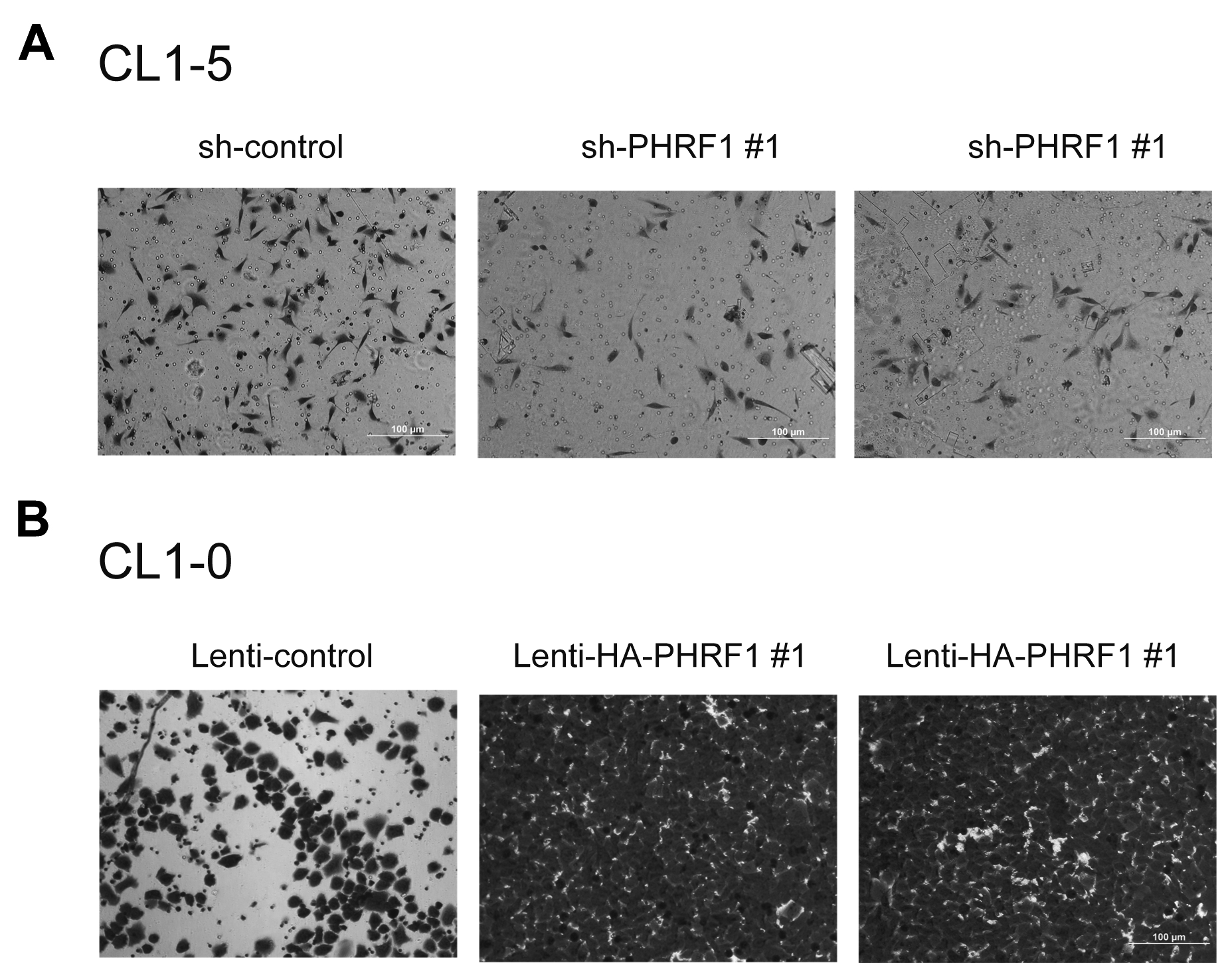

Supplement: S3 Fig — (A) 1x10^4^ control and PHRF1-depleted CL1-5 cells were seeded on the top well of a Boyden chamber in serum-free media, while culture medium supplemented with serum was placed in the well below for 24 h. Cells were photographed under phase-contrast microscopy and quantified. (B) 1x10^4^ control and PHRF1-overexpressing CL1-0 cells mixed with Matrigel were placed on the top of invasive chambers and allowed to penetrate to the lower surface of the filter. The cells on the lower surface of the membrane were stained with crystal violet and photographed under a light microscope. Scale bar, 100 μm. (TIF) [file pone.0236876.s003.tif]

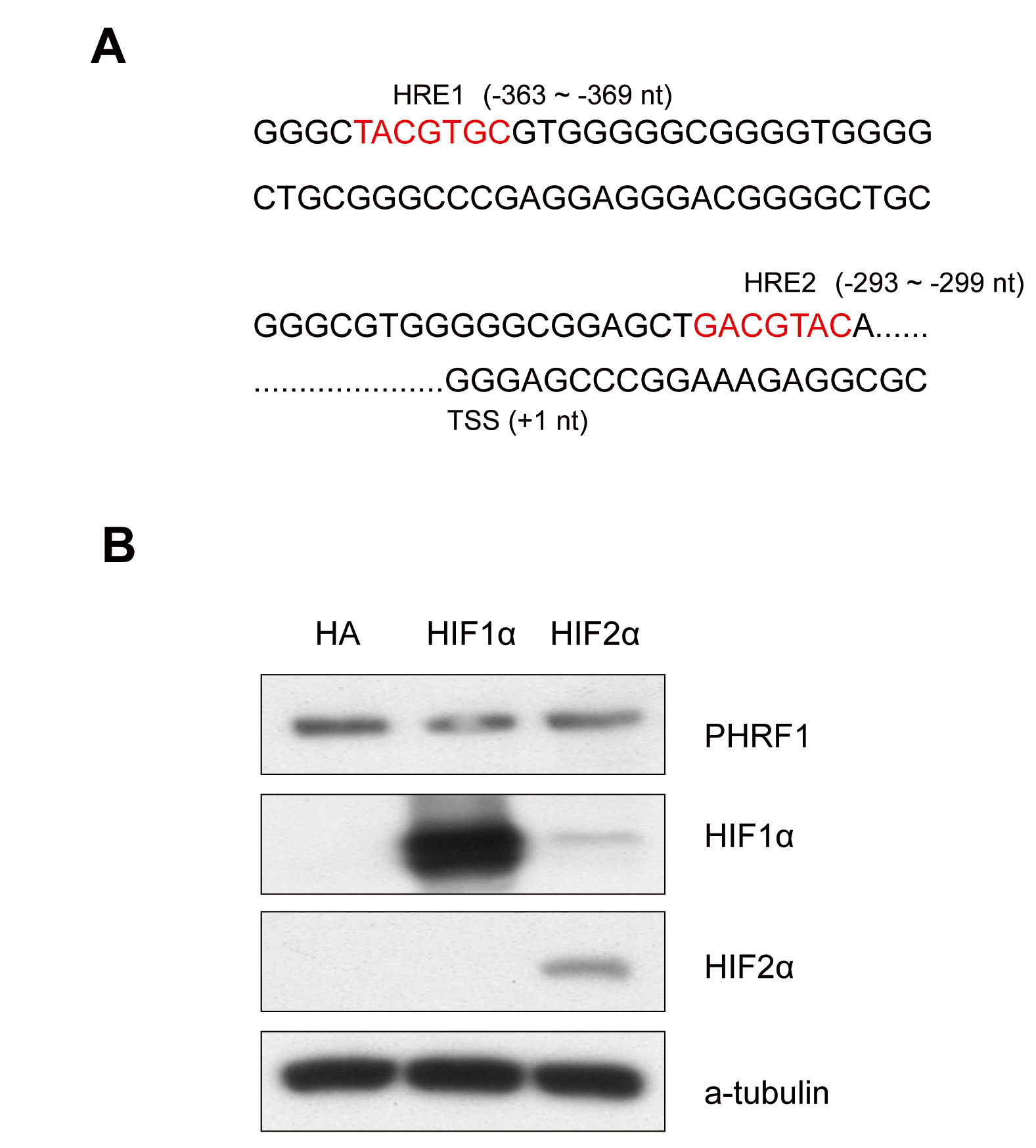

Supplement: S4 Fig — (A) Schematic representation of the proximal promoter (~ 350 nt upstream) of the PHRF1 gene. HRE: hypoxia response element. (B) Cell extracts prepared from HIF1α and HIF2α transfected HEK293T cells were immunoblotted with indicated antibodies. All Western blots were processed in identical conditions and cropped from S3 Raw images. (TIF) [file pone.0236876.s004.tif]

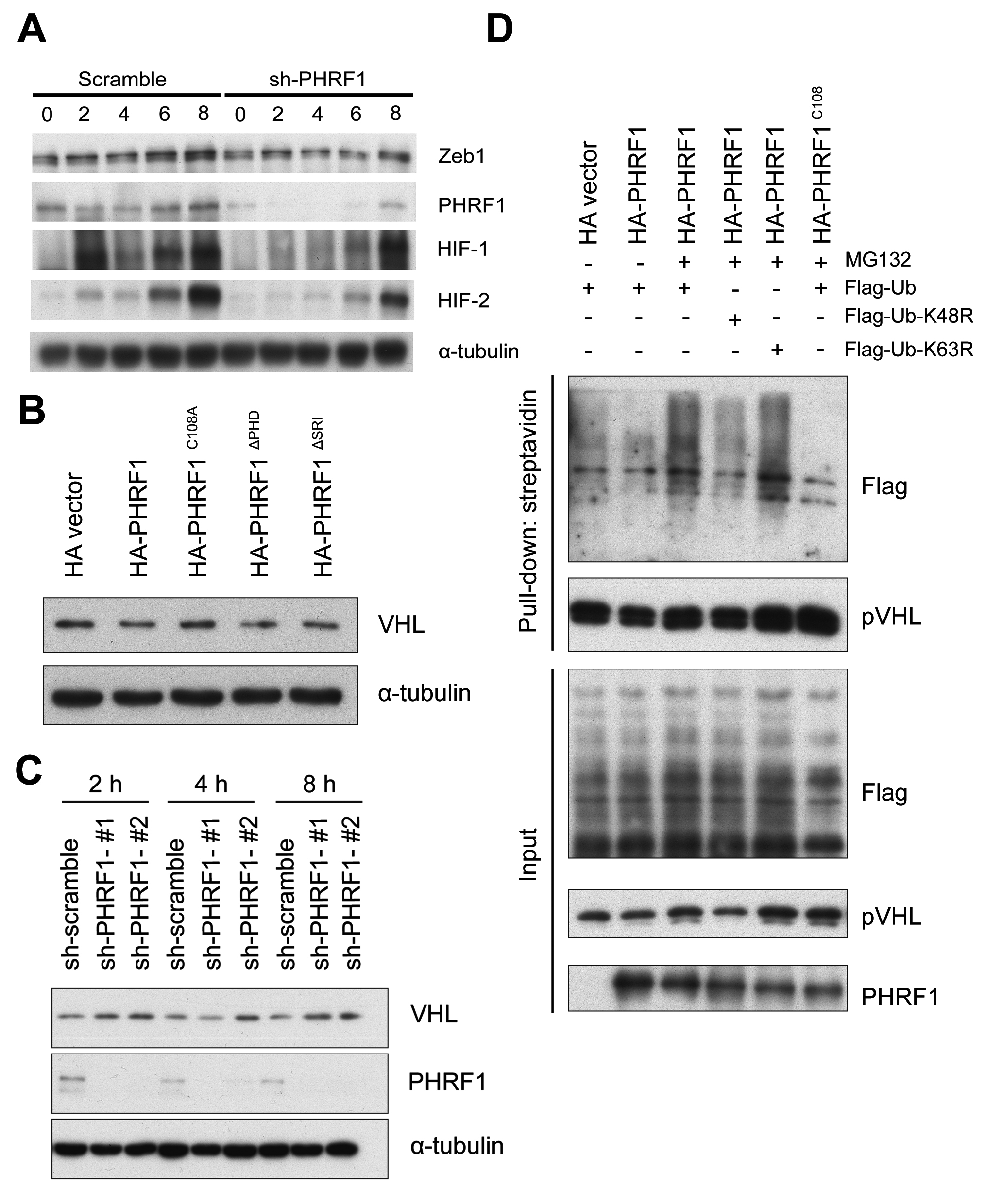

Supplement: S5 Fig — (A) Control and PHRF1-depleted A549 cells were placed in the hypoxia chamber (1% O2) for different time points and then immunoblotted with indicated antibodies. (B) Control and PHRF1 aberrant mutants were transfected into A549 cells and immunoblot analysis was carried out with indicated antibodies. (C) Control and PHRF1-depleted A549 cells were placed in the hypoxia chamber (1% O2) for different time points and then immunoblotted with anti-pVHL antibody. (D) HA-PHRF1 and HA-PHRF1C108A were co-transfected with FLAG-Ub and SBP-pVHL into HEK293T cells for 45 h and incubated with MG132 for another 3 h. Cell extracts were pulled down by streptavidin agarose and immunoblotted with indicated antibodies to detect ubiquitinated pVHL. All Western blots were processed in identical conditions and cropped from S3 Raw images. (TIF) [file pone.0236876.s005.tif]

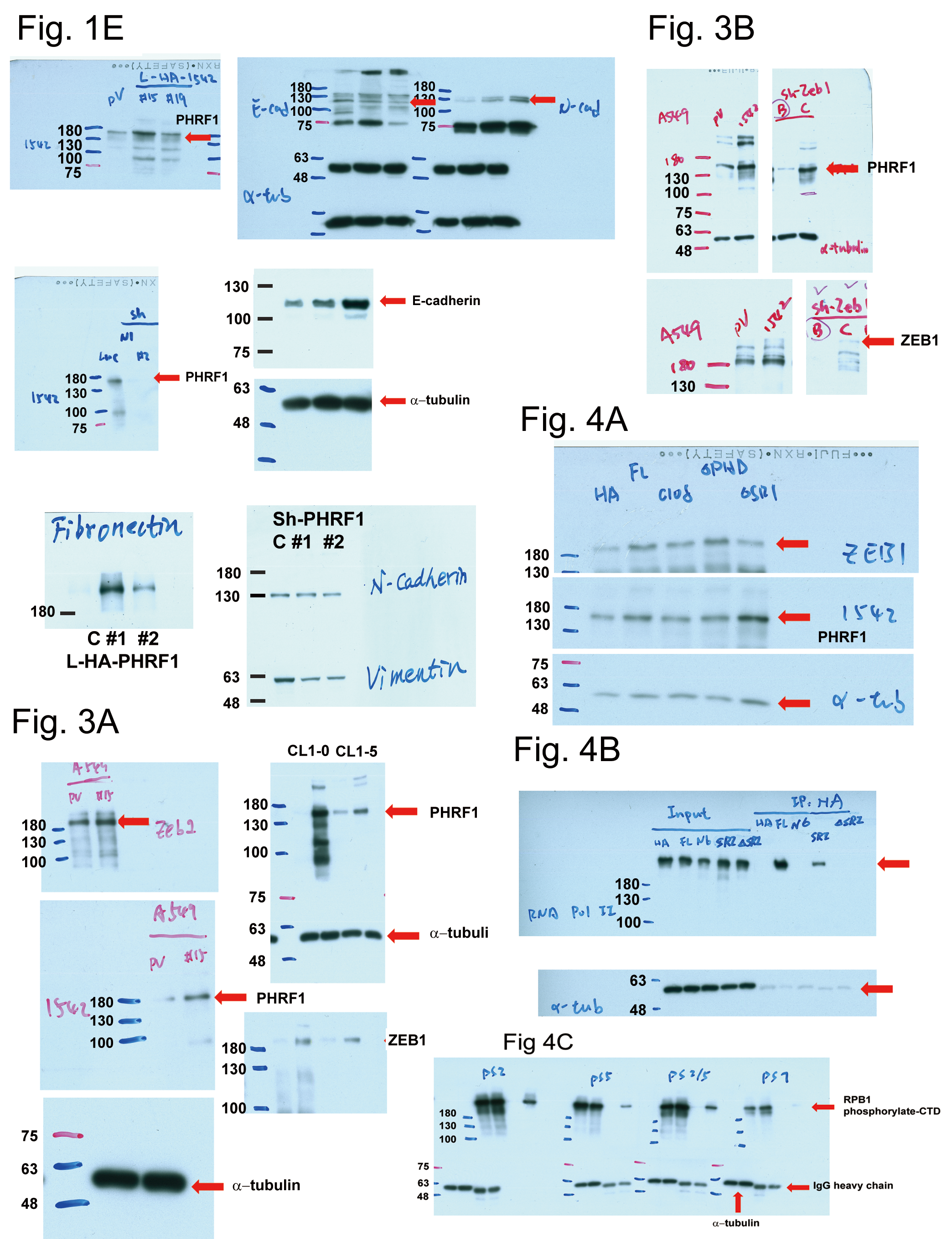

Supplement: S1 Raw images — (TIF) [file pone.0236876.s008.tif]

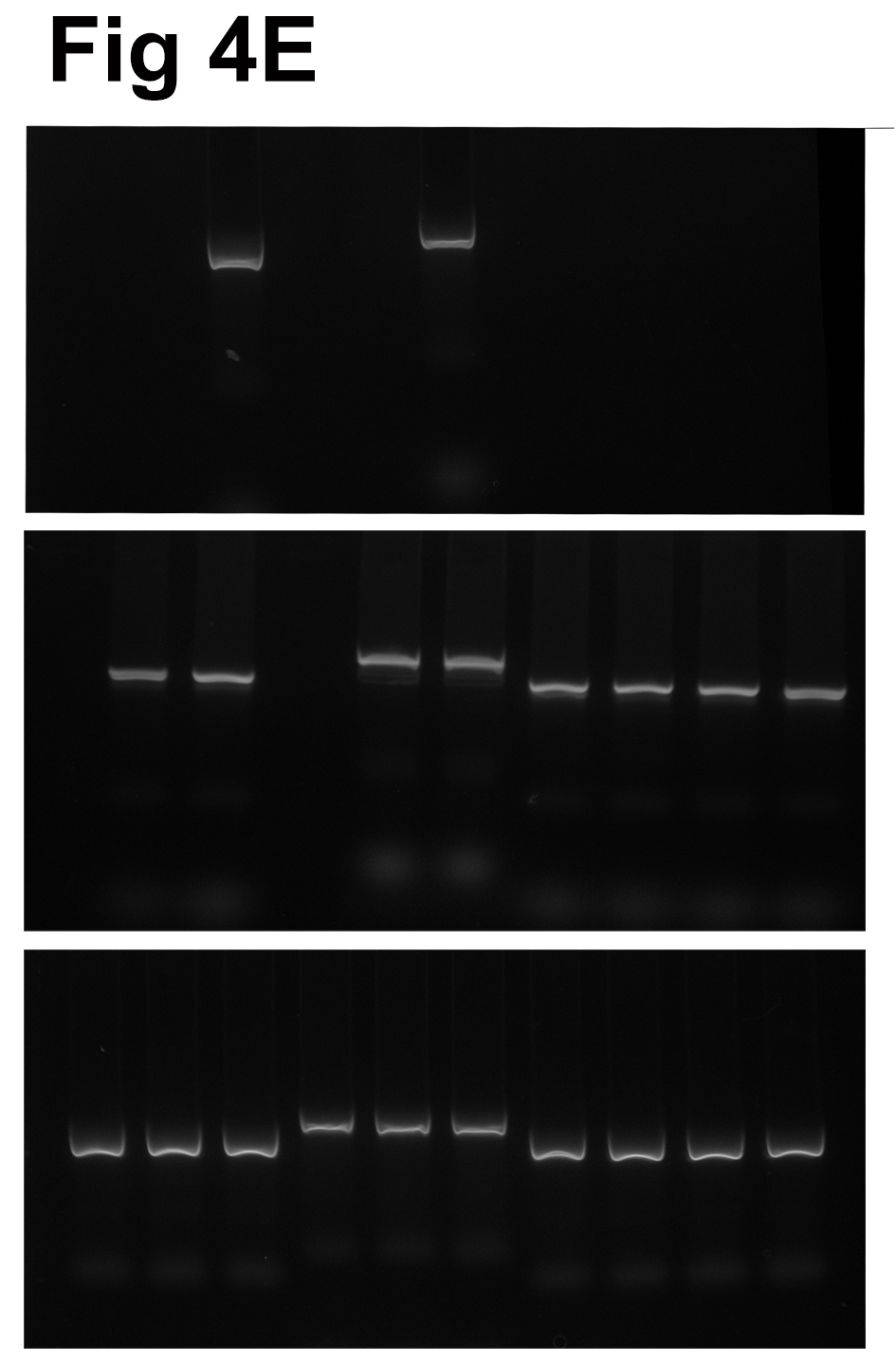

Supplement: S2 Raw images — (TIF) [file pone.0236876.s009.tif]

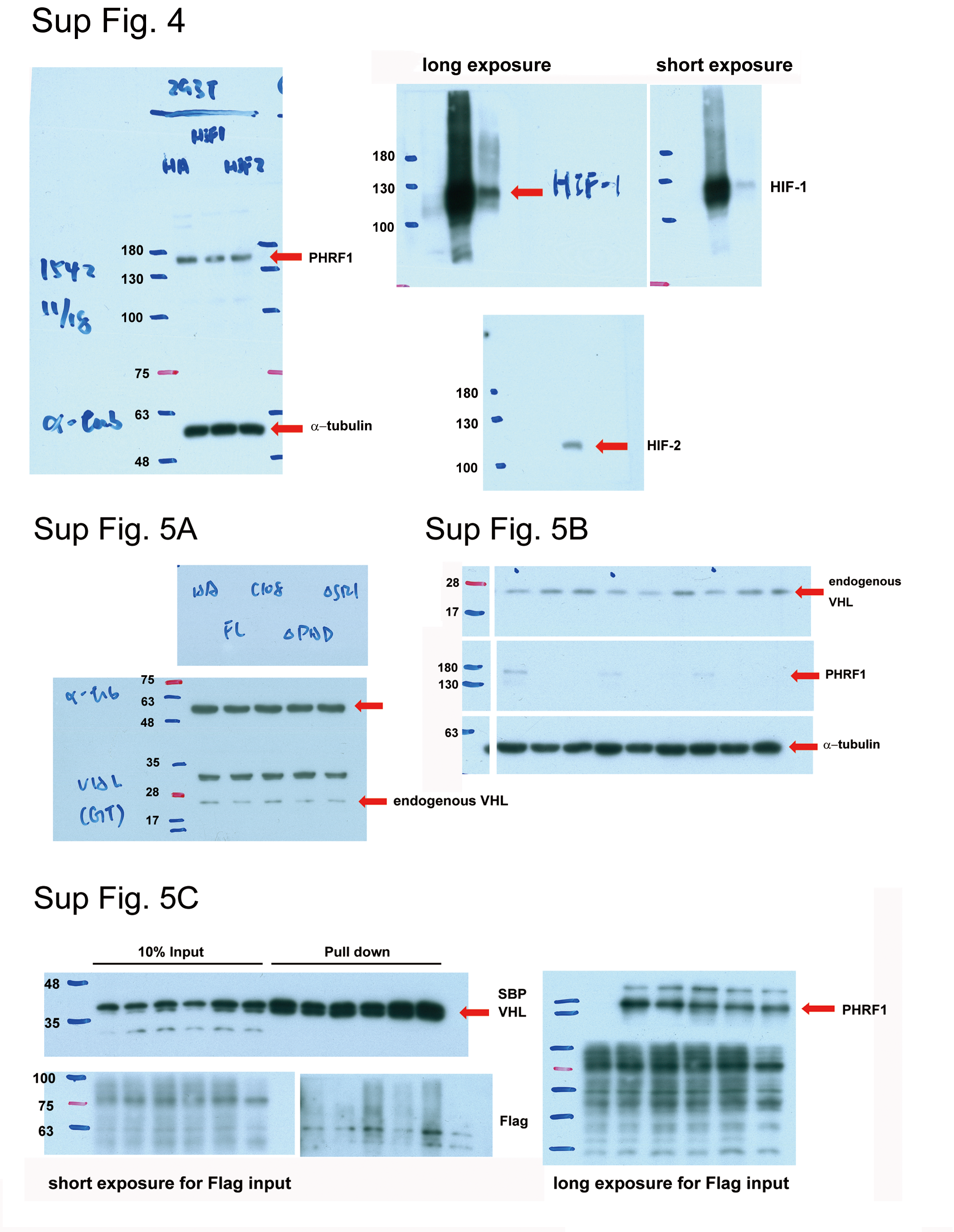

Supplement: S3 Raw images — (TIF) [file pone.0236876.s010.tif]
