# Supplementary material for: PHRF1 promotes migration and invasion by modulating ZEB1 expression
Source: PLoS One. 2020 Jul 30;15(7):e0236876. doi: 10.1371/journal.pone.0236876 (PMC7392320; doi:10.1371/journal.pone.0236876)
Supplement: S1 Table — (DOC) [file pone.0236876.s006.doc]

**S1 Table. Primer sequences of RT-qPCR.**

| **Gene** | **Forward primer (5’→3’)** | **Reverse primer (5’→3’)** |
| --- | --- | --- |
| ZEB1 | GGATGACAGAAAGGAAGGGCA | TCTGCATCTGACTCGCATTC |
| ZEB2 | AGCCTCTGTAGATGGTCCAG | GTCACTGCGCTGAAGGTACT |
| SNAI1 | AAGGCCTTCTCTAGGCCCTG | ATCTGAGTGGGTCTGGAGGT |
| PHRF1 | CGAGTGAAGAGAAGAGGGA | TCAACACTGACGGGATGCAG |
| GAPDH | TGACATCAAGAAGGTGGTGAAG | AGAGTGGGAGTTGCTGTTGAAG |
