# Supplementary material for: PHRF1 promotes migration and invasion by modulating ZEB1 expression
Source: PLoS One. 2020 Jul 30;15(7):e0236876. doi: 10.1371/journal.pone.0236876 (PMC7392320; doi:10.1371/journal.pone.0236876)
Supplement: S2 Table — (DOCX) [file pone.0236876.s007.docx]

**S2 Table. Primer sequences of ChIP**

| **Gene** | **Forward primer (5’→3’)** | **Reverse primer (5’→3’)** |
| --- | --- | --- |
| ZEB1 | CTCGAGCATTTAGACACAAGCG | CTGCCCAGCTCCCCCTGACTC |
| ZEB1_neg | ACTGAAGACGACTTTCTTGATAT | GTGCAGGACCTTAAGGCAAGA |
| ZEB2 | AAGCGTTTGCGGAGACTTCAAG | TCTGTAGGAGAGAGACGCGAGAGA |
| ZEB2_neg | CTGTGCTCAGCATCCTCAAAT | ACCAATTCAGAGCCCACTTCCC |
| SNAI1 | GAGTGGTTCTTCTGCGCTACT | TCAAGACCTTGGGCTGGGACT |
| GAPDH | TCAAGACCTTGGGCTGGGACT | TGGCGACGCAAAAGAAGATGC |
